# Accommodation and disability-specific differences in nutritional status of children with disabilities in Kathmandu, Nepal: A cross-sectional study

**DOI:** 10.1186/s12889-023-14999-z

**Published:** 2023-02-13

**Authors:** Krishna Prasad Sapkota, Akira Shibanuma, Ken Ing Cherng Ong, Junko Kiriya, Masamine Jimba

**Affiliations:** Department of Community and Global Health, Graduate School of Medicine, The University of Tokyo, Tokyo, Japan

**Keywords:** Disabled children, Malnutrition, Thinness, Growth Disorders, Pediatric obesity

## Abstract

**Background:**

Worldwide, more than 150 million children < 18 years live with disabilities. These children are more vulnerable to malnutrition regardless of institutional care that they receive, such as daycare or residential care. In Nepal, little is known about the status of malnutrition and factors associated with malnutrition among children with disabilities. This study was conducted to investigate the factors associated with malnutrition based on the types of disability and accommodation.

**Methods:**

This institution-based, cross-sectional study was conducted in 22 institutions in the Kathmandu Valley, Nepal. From these institutions, parents/guardians of all children with disabilities were recruited who were present there on the day of data collection. They were interviewed using a structured questionnaire. The questionnaire included questions on demographic characteristics, disability type and severity, accommodation place, feeding practices, and dietary patterns. The outcome variables, stunting, underweight, and obesity were measured using height-for-age, weight-for-age, and body mass index-for-age, respectively. A generalized linear model was used to investigate the factors associated with stunting and underweight, and multinomial logistic regression was used to identify the factors associated with overweight and obesity.

**Results:**

Among the 345 children with disabilities, 45% were stunted, 33% were underweight, 19% were thin, and 12% were overweight. Children with physical disabilities (relative risk ratio = 1.88, 95% confidence interval [CI] = 1.26–2.81) were more likely to be stunted than those with sensory disabilities. Children with autism (adjusted odds ratio [aOR] = 5.56, 95% CI: 1.23–25.23) and intellectual disabilities (aOR = 5.84, 95% CI: 1.59–21.51) were more likely to be overweight and obese than those with sensory disabilities. No evidence was found regarding an association between accommodation type and malnutrition.

**Conclusion:**

Children with disabilities are vulnerable to malnutrition in several ways. Different types of disabilities are associated with different forms of malnutrition. Considering the types of disabilities, tailor-made approaches should be adopted to improve malnutrition status.

**Supplementary Information:**

The online version contains supplementary material available at 10.1186/s12889-023-14999-z.

## Background

The World Health Organization (WHO) estimated that over one billion global population has some form of disability, which is 15% of the global population [1]. Globally, 150 million children aged < 18 years have moderate-to-severe disabilities [2, 3]. Many children with disabilities (CWDs) reside in low- and middle-income countries (LMICs) [3, 4], and they are the most marginalized and vulnerable in society, lacking access to safe water and wholesome food [5].

Malnutrition and disability in children are major public health problems [6], as malnourished CWDs are more vulnerable to severe illnesses [7, 8] and deaths [9]. CWDs are more prone to malnutrition because of inadequate nutritional intake due to physical and neurological impairment, unmet need for nutrients, and less absorption of nutrients from the intestine [10, 11]. For these reasons, the Convention on the Rights of the Child and Convention on the Rights of Persons with Disabilities have advocated for proper nutrition of the disabled population of children and adults since 1990 [12, 13].

Many CWDs are kept in institutional care such as daycare or residential care [14]. In the institutional setting, nutritious meals are usually provided, and it is a promising intervention to reduce the risk of malnutrition if appropriately managed [15]. Among children without disabilities, similar meals can improve food diversity and the intake of micronutrients and protein [16, 17]. However, among CWDs, malnutrition is prevalent in institutional settings [14, 18, 19], and there are significant gaps across and within countries when it comes to institutional nutrition services for children in LMICs [15].

As assessing nutritional status is difficult in CWDs, it is often not appropriately evaluated [20]. Moreover, in institutional settings, studies on malnutrition and disability in LMICs are limited, particularly in Nepal. Furthermore, the factors contributing to malnutrition could differ among CWDs living in institutions and in their homes.

This study was conducted to investigate the factors associated with malnutrition among CWDs according to the types of disability and accommodation.

## Methods

### Study design and settings

This was a cross-sectional study examining malnutrition status among CWDs and was conducted in all three districts of the Kathmandu Valley of Nepal, namely Kathmandu, Lalitpur, and Bhaktapur, from August to September 2019. The target population consisted of CWDs attending institutions, such as special schools, daycare centers, or rehabilitation homes in the study sites and their parents/guardians.

### Study participants

CWDs from 5 to 17 years of age were eligible, but they were excluded if they could not stand with any assistance or were wheelchair users, as a different technique had to be applied to capture the height for those people to measure stunting.

As the official list of the institutions for CWDs was not available, a list of related institutions (including special schools, daycare centers, hostels, and rehabilitation homes) of Kathmandu Valley was collected along with contact information from the Ministry of Women, Children and Senior Citizen, National Federation of the Disabled-Nepal (NFDN), and Karuna Foundation Nepal (KFN). The NFDN is a national body for persons with disabilities, and the KFN is a leading non-governmental organization for CWDs in Nepal. After obtaining the list, telephone contact and email communication were conducted with the institution’s contact person for permission to conduct data collection. Twenty-seven institutions were approached, and 22 provided permission to conduct the survey. On the date of data collection, all eligible children present in the institution were enumerated and measured for anthropometry in all the 22 institutions.

### Outcome variables

#### Nutritional status

The following growth indicators were calculated to measure the level of malnutrition: height, weight, and body mass index (BMI). All indicators were standardized with z-scores, and height for age z-score (HAZ), weight for age z-score (WAZ), and BMI for age z-score (BAZ) were calculated using the WHO’s child growth standards. WHO cutoffs were then applied to distinguish between malnourished and healthy children. Overweight and obesity were calculated by BAZ, stunting by HAZ, and underweight by WAZ using the WHO reference 2007 [21]. The WHO standard cutoffs were used to categorize all growth indicators: overweight (BAZ > + 1 Standard Deviation (SD) and ≤ + 2 SD), obesity (BAZ > + 2SD), thinness (BAZ <-2 SD), stunting (HAZ < -2SD), and underweight (WAZ< -2SD). Z-values below − 3SD were used to define severely malnourished as severely stunted, severely underweight, severely obese, or severely thin [22].

### Exposure variables

#### Disability

The Government of Nepal has classified disabilities into ten different types: physical, vision-related, hearing-related, vocal and speech-related, deaf-blind, intellectual, mental and psychosocial, hemophilia, autism, and multiple disabilities, and the Government of Nepal issued a disability ID card for them. Children’s disability types were then verified with the notes of medical doctors. As this study was institution-based, all children had medical notes from their doctors. However, due to less data from some disability types, disability types were re-categorized. Finally, disability was categorized into the following types: physical disability, sensory (vision-related, hearing-related, vocal and speech-related, and deaf-blind) disability, developmental (intellectual, mental, and psychosocial) disability, autism, and multiple disabilities. Furthermore, none of the children with hemophilia were interviewed.

#### Accommodation

Places of accommodation were categorized as either homes or institutions. For example, children sleeping in the hostels of special schools or rehabilitation homes were categorized as having institutional accommodations. Children staying at an institution only for daytime and sleeping at home with family were categorized as accommodated at home.

### Covariates

Information on the children’s age, sex, parental status, feeding ability, feeding diversity, and severity of disability was collected. Age was treated as a continuous variable. Parental status was dichotomized, either orphaned or not. Feeding ability was dichotomized if a child was self-fed or needed feeding support. Children’s dietary diversity score (CDDS) was calculated by asking 24-hours and seven-days recall questionnaire [8]. Thirteen types of food categories were used for 24-hours and seven-days recall questions. Seven food categories were used to calculate CDDS: starchy staples, pulses and legumes, eggs, dairy products, meats, vitamin A-rich vegetables, and other fruits and vegetables [23]. Disability severity was assessed using the disability ID card of each CWD. The Government of Nepal provides four different color cards for CWDs, based on their severity. For example, the red card is given to children with profound disabilities, the blue card is given to children with severe disabilities, the yellow card is given to children with moderate disabilities, and the white card is given to children with mild disabilities [24].

### Data collection

Primary data were collected using a structured questionnaire and anthropometric measurements of children aged 5 to 17 years. For anthropometric measurements, the children’s height and weight were measured. Weight was measured using a standard digital weight scale, Seca (Model no. 874, Germany), and height was measured using a wall-mountable Bioplus Stature Meter (Model no. 26 M/1,013,522, India). The measurement of weight was recorded to the nearest 0.1 kg and height to the nearest 0.1 cm. Other information was collected by administering a questionnaire to the parents or caretakers of the children or staff at that institution.

Face-to-face or telephone interviews were conducted in the Nepali language to collect the data from parents/caretakers. The questionnaire to the institutional staff included information about the types of disability, overnight stay of CWDs, and socio-demographic information. The parents/caretakers’ included feeding practices, dietary patterns, and maternal and child health-related information. The questionnaire was drafted in this study based on Nepali translation from the existing English questionnaire used to assess malnutrition among children with disabilities in Kenya [8]. To ensure its validity, the questionnaire was modified to fit the local context. For example, the food names in the 24-hour recall questions were reviewed so that parents/caretakers could understand them. The first author did all interviews and anthropometric measurements.

### Data analysis

The collected data were analyzed to examine hypotheses that the associations existed between exposures, namely disability type and accommodation, and malnutrition outcomes, after adjusting for various covariates mentioned above. A generalized linear model (GLM) was used for HAZ and WAZ outcomes. In the model, relative risk ratio (RRR) was estimated for the sizes of associations. In GLM, the Poisson distribution and the log link function were used to fit the model, and a robust standard error was used for variance estimates. A multinomial logistic regression model was used for BAZ data. In the model, odds ratio was estimated for the sizes of associations. In the model, BAZ was categorized into three labels, namely, underweight, normal and overweight/obesity; normal was the reference outcome category to present odds ratio regarding underweight and overweight/obesity. Unadjusted and adjusted results were presented from both GLM and multinomial logistic regression model. In the main analyses, the adjusted models included accommodation as an exposure variable, in addition to children’s age, sex, parental status, feeding ability as covariates. When a parent or caregiver did not remember the food name of meals, feeding diversity score was treated as missing. When they did not present the disability ID card or did not remember its color, severity of disability was treated as missing. Therefore, feed diversity and severity of disability were not included in the main analyses. Instead, separate analyses were conducted for the models including these variables and the variables from the main analysis by using the subsamples that included CWDs without missing variables. Data were entered into Excel 2013, and Z-score values for anthropometric measurements were calculated using WHO Anthro Plus software. Descriptive and inferential data analyses were performed using Stata version 15.0 (StataCorp. 2017. College Station, TX: StataCorp LLC.).

### Ethics

Ethical clearance was obtained from the Research Ethics Committee of the University of Tokyo (Serial no. 2019090NI) and the Ethical Review Board of the Nepal Health Research Council (Reg. no. 396/2019). Approval letters from the Ministry and NFDN were obtained prior to data collection. Each institution approved data collection in the field. Written informed consent was obtained from the parents or caretakers of all CWDs. Written assent was also obtained from all CWDs. Participation was voluntary, and the confidentiality of the respondents was maintained.

## Results

Data were collected from 22 institutions and the parents/guardians of all CWDs were recruited on the day of data collection. A total of 466 eligible CWDs were potential participants in these institutions; however, 345 were present on the day of data collection.

### Socio-demographic characteristics of children with disabilities

Among the 345 CWDs participating in the study, 48.4% went to institutions for daytime only and slept at home at night, and 51.6% stayed at institutions for full-time institutional care. Table 1 shows that 61.7% of children were boys, and the number of children who stayed overnight at institutions was also higher among boys (65.2%). The mean age of the children was 11.3 [SD 3.6] years.

**Table 1:** Socio-demographic characteristics of children with disabilities (*n* = 345)

	**Home accommodation (** ***n*** **=167 children)** **n (%)**	**Institutional accommodation (** ***n*** **=178 children)** **n (%)**	***p*** **-value**
**Sex**			
Boys	97 (58.1)	116 (65.2)	0.176
Girls	70 (41.9)	62 (34.8)	
**Age**			
5–9 years	73 (43.7)	40 (22.5)	<0.001
10–14 years	70 (41.9)	80 (44.9)	
15–17 years	24 (14.4)	58 (32.6)	
**Parental status**			
Orphan	0 (0.0)	29 (16.3)	<0.001
Not Orphan	167 (100.0)	149 (83.7)	
**Disability type**			
Physical	25 (25.0)	78 (43.8)	<0.001
Sensory	13 (7.8)	47 (26.4)	
Developmental	70 (41.9)	39 (21.9)	
Autism	23 (13.8)	9 (5.1)	
Multiple disability	36 (21.6)	5 (2.8)	
**Disability ID card (** ***n*** **=334)**			
Yes	105 (64.4)	126 (73.7)	0.067
No	58 (35.6)	45 (26.3)	
**Type of disability ID card (** ***n*** **=230)**			
Red card (Profound disability)	69 (65.7)	27 (21.6)	<0.001
Blue card (Severe disability)	32 (30.5)	63 (50.4)	
Yellow card (Moderate disability)	3 (2.9)	26 (20.8)	
White card (Mild disability)	1 (1.0)	9 (7.2)	
**Methods of communication**			
Verbal	82 (49.1)	125 (70.2)	<0.001
Non-verbal	85 (50.9)	53 (29.8)	
**Feeding ability of children**			
Feeds self	118 (70.7)	170 (95.5)	<0.001
Needed feeding	49 (29.3)	8 (4.5)	
**CDDS (** ***n*** **=333)**			
≤4	30 (19.0)	12 (6.9)	<0.001
5	49 (31.0)	46 (26.3)	
6	51 (32.3)	104 (59.4)	
7	28 (17.7)	13 (7.4)	

*CDDS* Children’s dietary diversity score

The type of disability differed substantially between CWDs who stayed overnight at home and at institutions. Among CWDs who stayed overnight at home, 41.9% had a developmental disability, followed by a physical disability (25.0%). Among CWDs who stayed overnight at institutions, 43.8% had a physical disability, followed by a sensory disability (26.4%). Only 69% of CWDs possess a disability ID card. Furthermore, 13% of children ate four or fewer varieties of food per week (Table 1).

### Nutritional status of children with disabilities

Table 2 shows the nutritional status of CWDs by accommodation type. More than 60% of the children sleeping at home and at institutions had a normal BMI for their age. Overweight (10.2%) and obesity (4.2%) were higher among CWDs sleeping at home. In the height-for-age category, a little more than 50% of children sleeping at home and institution had a normal height for their age. Severe stunting was higher among children sleeping in an institution, and stunting was higher among CWDs sleeping at home. In the malnutrition type by weight category, almost 20% of CWDs sleeping at home and at an institution were underweight and severely underweight, respectively.

Overall, more than 40% of CWDs were stunted, 33.3% were underweight, and 12.2% were overweight and obese. More than 60% of CWDs had at least one form of malnutrition.

**Table 2:** Nutritional status of children with disabilities

	Type	Home accommodation n (%)	Institutional accommodation n (%)	Total n (%)
**Malnutrition type by BMI-for-age (** ***n*** **=345)**	Severe thinness	19 (11.4)	12 (6.7)	31 (9.0)
	Thinness	16 (9.6)	20 (11.2)	36 (10.4)
	Normal	107 (64.1)	129 (72.5)	236 (68.4)
	Overweight	17 (10.2)	15 (8.4)	32 (9.3)
	Obesity	7 (4.2)	1 (0.6)	8 (2.3)
	Severely obese	1 (0.6)	1 (0.6)	2 (0.6)
**Malnutrition type by height-for-age (** ***n*** **=345)**	Severely stunted	31 (18.7)	42 (23.6)	73 (21.1)
	Stunted	42 (25.2)	39 (21.9)	81 (23.5)
	Normal	94 (56.3)	97 (54.5)	191 (55.4)
**Malnutrition type by weight-for-age** ^a^ ** (** ***n*** **=117)**	Severely underweight	10 (13.3)	9 (21.4)	19 (16.2)
	Underweight	14 (18.7)	6 (14.3)	20 (17.1)
	Normal	51 (68.0)	27 (64.3)	78 (66.7)
**Any form of malnutrition**	Yes	105 (62.9)	111 (62.4)	216 (62.6)
	No	62 (37.1)	67 (37.6)	129 (37.4)

*BMI* Body Mass Index

^a^ Calculations performed for children below 10 years of age

### Association of disability type and accommodation with stunting and underweight

Table 3 shows that disability types and accommodation were not associated with undernutrition, adjusted for other covariates, including feeding ability, dietary diversity and the severity of disability. Among other covariates, the risk of being underweight was higher among children that required feeding by their parents/caretakers (RRR = 1.87, 95% confidence interval [CI] = 1.26–2.77) than those who could feed themselves. A high CDDS was associated with a decreased risk of being underweight (RRR = 0.83, 95% CI = 0.69–0.99). Children with red disability ID cards were more likely to be underweight (RRR = 3.14, 95% CI = 1.27–7.83) than those with white- and yellow-colored cards.

**Table 3:** Factors associated with being underweight (*n* = 117)

**Variables **	**Unadjusted RRR**	**95% CI**	***p*** **-value**	**Adjusted RRR**	**95% CI**	***p*** **-value**
*Main analysis *						
**Disability type**						
Sensory	Reference					
Physical	2.10	1.01 – 4.35	0.047	1.72	0.77 – 3.84	0.183
Developmental	1.62	0.87 – 3.03	0.128	1.24	0.61 – 2.53	0.560
Autism	0.42	0.07 – 2.48	0.336	0.32	0.05 – 2.09	0.236
Multiple disabilities	2.37	1.34 – 4.19	0.003	1.48	0.76 – 2.90	0.251
**Accommodation**						
Home	Reference					
Institution	1.12	0.71 – 1.75	0.631	1.11	0.57 – 2.13	0.754
**Age**	1.00	0.82 – 1.21	1.000	0.98	0.79 – 1.19	0.821
**Sex**						
Boy	Reference					
Girl	0.64	0.34 – 1.20	0.163	0.62	0.35 – 1.09	0.103
**Parental status**						
Not orphan	Reference					
Orphan	2.11	1.09 – 4.11	0.027	1.50	0.57 – 3.96	0.410
**Feeding ability of a child**						
Self-fed	Reference					
Needed feeding	1.81	1.27 – 2.59	0.001	1.87	1.26 – 2.77	0.002
*Analysis excluding missing data* ^a^						
**CDDS (*n*=112)**	0.82	0.65 – 1.02	0.081	0.83	0.69 – 0.99	0.041
**Types of disability ID card (*n*=67)**						
White & Yellow card (Mild-Moderate disability)	Reference					
Red card (Profound disability)	2.03	0.86 – 4.78	0.104	3.14	1.26 – 7.83	0.014
Blue card (Severe disability)	1.40	0.56 – 3.50	0.472	2.33	0.78 – 6.95	0.130

*CDDS* Children’s dietary diversity score, *RRR* Relative risk ratio, *CI* Confidence interval

^a^ Variables in the main analysis were adjusted (the results of these variables are not reported).

Table 4 presents the factors associated with stunting. Children with physical disabilities (RRR = 1.88, 95% CI = 1.26–2.81) were more likely to be stunted than those with sensory disabilities. Accommodation was not associated with stunting. Among other covariates, children holding red (RRR = 1.67, 95% CI = 1.26–2.23) and blue-colored disability ID cards (RRR = 1.49, 95% CI = 1.27–1.75) were associated with stunting compared to those holding other disability ID cards. Age (RRR = 1.06, 95% CI = 1.03–1.09), parental status (RRR = 1.52, 95% CI = 1.13–2.03), and feeding ability of a child (RRR = 1.71, 95% CI = 1.33–2.19) were associated with stunting.

**Table 4:** Factors associated with stunting (*n* = 345)

**Variables **	**Unadjusted RRR**	**95% CI**	***p*** **-value**	**Adjusted RRR**	**95% CI**	***p*** **-value**
*Main analysis*						
**Disability type**						
Sensory	Reference					
Physical	1.97	1.32 – 2.94	0.001	1.88	1.26 – 2.81	0.002
Developmental	1.44	0.96 – 2.15	0.079	1.23	0.81 – 1.86	0.333
Autism	1.04	0.45 – 2.43	0.925	1.03	0.45 – 2.37	0.945
Multiple disabilities	1.46	0.91 – 2.36	0.119	1.11	0.65 – 1.89	0.689
**Accommodation**						
Home	Reference					
Institution	1.04	0.78 – 1.38	0.783	0.87	0.72 – 1.05	0.154
**Age**	1.05	1.01 – 1.08	0.010	1.06	1.03 – 1.09	<0.001
**Sex**						
Boy	Reference					
Girl	1.12	0.92 – 1.35	0.262	1.08	0.93 – 1.26	0.313
**Parental status**						
Not orphan	Reference					
Orphan	1.44	1.10 – 1.90	0.009	1.52	1.13 – 2.03	0.005
**Feeding ability of a child**						
Self-fed	Reference					
Needed feeding	1.43	1.07 – 1.92	0.017	1.71	1.33 – 2.19	<0.001
*Analysis excluding missing data* ^a^						
**CDDS (*n*=333)**	1.01	0.86 – 1.17	0.952	0.95	0.83 – 1.10	0.520
**Types of disability ID card (*n*=230)**						
White & Yellow Card (Mild-Moderate disability)	Reference					
Red card (Profound disability)	1.35	1.01 – 1.80	0.044	1.67	1.26 – 2.23	<0.001
Blue card (Severe disability)	1.00	0.74 – 1.35	0.997	1.49	1.27 – 1.75	<0.001

*CDDS* Children’s dietary diversity score, *RRR* Relative risk ratio, *CI* Confidence interval

^a^ Variables in the main analysis were adjusted (the results of these variables are not reported).

### Association of disability type and accommodation with overweight, obesity and thinness

Table 5 depicts factors associated with overweight/obesity and thinness. Children with autism (adjusted odds ratio [aOR] = 5.56, 95% CI = 1.23–25.23) and intellectual disabilities (aOR = 5.84, 95% CI = 1.59–21.51) were more likely to be overweight and obese. Accommodation was not associated with overweight/obesity nor thinness. Among other covariates, age (aOR = 1.14, 95% CI = 1.05–1.25) and children’s feeding ability (aOR = 2.81, 95% CI = 1.26–6.24) were associated with thinness. CDDS was associated with a lower risk of thinness (aOR = 0.67, 95% CI = 0.47–0.94).

**Table 5:** Factors associated with overweight and thinness (*n* = 345)

	**Overweight/Obesity**				**Thinness**			
**Variables**	**Unadjusted OR (95% CI)**	***p*** **-value**	**Adjusted OR ** **(95% CI)**	***p*** **-value**	**Unadjusted OR (95% CI)**	***p*** **-value**	**Adjusted OR (95% CI)**	***p*** **-value**
*Main analysis*								
**Disability type**								
Sensory	Reference							
Physical	1.29 [0.31 – 5.40]	0.728	1.32 [0.31 – 5.56]	0.702	1.69 [0.69 – 4.12]	0.247	1.68 [0.68 – 4.19]	0.262
Developmental	5.78 [1.64 – 20.35]	0.006	5.84 [1.59 – 21.51]	0.008	1.98 [0.81 – 4.84]	0.135	1.79 [0.68 – 4.68]	0.234
Autism	5.44 [1.28 – 23.11]	0.022	5.56 [1.23 – 25.23]	0.026	1.17 [0.32 – 4.30]	0.817	1.07 [0.27 – 4.22]	0.920
Multiple disabilities	1.96 [0.37 – 10.42]	0.430	2.15 [0.36 – 12.87]	0.400	3.18 [1.17 – 8.69]	0.024	2.53 [0.79 – 8.18]	0.120
**Accommodation**								
Home	Reference							
Institution	0.56 [0.29 – 1.10]	0.093	0.81 [0.36 – 1.78]	0.600	0.76 [0.44 – 1.31]	0.319	0.87 [0.44 – 1.72]	0.693
**Age**	1.05 [0.96 – 1.15]	0.311	1.07 [0.96 – 1.19]	0.190	1.08 [0.99 – 1.16]	0.064	1.14 [1.05 – 1.25]	0.003
**Sex**								
Boy	Reference							
Girl	1.03 [0.53 – 2.00]	0.937	1.19 [0.59 – 2.43]	0.623	0.69 [0.39 – 1.23]	0.208	0.61 [0.33 – 1.12]	0.113
**Feeding ability of a child**								
Self-fed	Reference							
Needed feeding	0.83 [0.30 – 2.27]	0.718	0.79 [0.26 – 2.43]	0.687	2.43 [1.28 – 4.65]	0.007	2.81 [1.26 – 6.24]	0.011
*Analysis excluding missing data* ^a^								
**CDDS (*n*=333)**	0.71 [0.49 – 1.03]	0.902	0.77 [0.52 – 1.15]	0.206	0.72 [0.53 – 0.97]	0.034	0.67 [0.47 – 0.94]	0.021
**Types of disability ID card (*n*=230)**								
White & Yellow card (Mild-Moderate disability)	Reference							
Red card (Profound disability)	4.33 [0.92 – 20.23]	0.063	2.37 [0.39 – 14.23]	0.345	2.39 [0.93 – 6.12]	0.069	2.79 [0.87 – 8.87]	0.082
Blue card (Severe disability)	2.29 [0.48 – 10.97]	0.299	2.03 [0.34 – 11.93]	0.434	0.71 [0.26 – 1.99]	0.520	0.77 [0.22 – 2.67]	0.684

*CDDS* Children’s Dietary Diversity Score, *CI* Confidence Interval, *OR* Odds Ratio

^a^ Variables in the main analysis were adjusted (the results of these variables are not reported).

## Discussion

The major findings of this study are as follows. First, all forms of malnutrition were pervasive in CWDs, and disability types were associated with stunting, overweight, and obesity. Specifically, physical disabilities were associated with stunting, and autism and developmental disabilities were associated with overweight and obesity. Second, the accommodation type was not associated with any form of malnutrition. Third, among other factors, the feeding ability of a child was associated with underweight, stunting, and thinness, while age was associated with stunting and thinness. Similarly, being an orphan was associated with stunting.

In this study, more than 60% of CWDs suffered from at least one form of malnutrition. Compared with non-disabled adolescent students in the Kaski and Dang districts of Nepal, malnutrition status was worse among CWDs in this study [25, 26]. Nepal Demographic and Health Survey (NDHS) 2016 shows that 36% of children under-5 years were stunted, 27% were underweight, and 1% were overweight [27]. Compared with children in the NDHS data, CWDs in Kathmandu valley had higher prevalence of all forms of malnutrition. More than 12% of CWDs were overweight, which was higher than 1% in the NDHS data. This could be because of CWD’s sedentary lifestyle. Children with disabilities are also more susceptible to malnutrition in Kenya, and CWDs are twice as likely to have undernutrition compared with those without disabilities [8]. In a systematic review conducted in 2018, CWDs were three times more likely to be underweight and twice as likely to be stunted than non-disabled children in LMICs [28]. In Turkey [29] and the US [30, 31], overweight and obesity were also higher among CWDs than among non-disabled children. Malnutrition is a serious concern in CWDs.

Among the different types of undernutrition, stunting was high among children with physical disabilities. Stunting was also increased among children with physical disabilities in Iran [32] and Bangladesh [6]. In Iran, children with physical disabilities have inadequate nutritional intake, and the majority of them do not meet the minimum dietary requirements for different micronutrients [32]. In this study, age, being an orphan, and not being able to feed by themselves were associated with stunting. Increasing age was also considered a risk factor for stunting among non-disabled school-age children in Ethiopia [33] and Bangladesh [34]. Stunting is the result of chronic malnutrition that can be observed in later childhood. Furthermore, orphaned children could have a paucity of a balanced diet, as they are deprived of their parents’ love and care, which may have resulted in stunting.

Developmental disabilities and autism were associated with being overweight or obese in this study compared to other types of disabilities. Similar results were observed in reports from Chile [35], Mexico [36], and Australia [37] and in systematic reviews [38–40], where developmental disability was associated with obesity. Furthermore, the study findings are comparable with those of other studies that have found a link between autism [41, 42] and being overweight or obese. However, Kilinc et al. [29] showed no association between disability type and being overweight or obese. Low physical exercise, unhealthy diet, and low social participation could be the causes of obesity among children with developmental disabilities. Children are hindered from participating in sports, leisure, and recreational activities because of their disability, resulting in fat deposition in the body. Children with certain developmental disabilities also tend to eat high-energy foods and are eager to overeat [43].

In the bivariate analysis in this study, overweight and obesity was higher among children sleeping at home, whereas stunting and underweight were slightly higher among children sleeping at an institution. However, the accommodation type was not significantly associated with malnutrition after controlling for other factors (sex, age, disability type, and feeding ability). The family’s decision on accommodation type might have been based on the type and severity of disability and the socioeconomic characteristics of the children’s family. In the study site, children with developmental and multiple disabilities and children with a red color disability ID card (who are profoundly disabled) tended to accommodate less at an institution than at home. Therefore, these disability characteristics might explain malnutrition rather than accommodation type, according to the multivariable analysis results in this study.

Feeding ability and CDDS were significantly associated with underweight and thinness in this study. Feeding ability may result in insufficient nutrient intake due to difficulty eating and swallowing food [44] and when the management and care of CWDs are insufficient, this too may lead to insufficient nutrient intake [19]. Lack of dietary diversity was associated with being underweight in South Africa [45] and stunting in a multi-country study [45, 46]; however, this study did not find evidence of an association between CDDS and stunting. This might be because dietary variety data were obtained only from the previous week, and stunting is a long-term cumulative process. In this study, orphaned CWDs living in an institution were associated with stunting and wasting [47, 48]. This could be because orphans solely relied on the institution for food, and the sanitation of the institution might have played a role in undernutrition.

The results from this study imply that CWDs should receive quality interventions that mitigate their malnutrition. The prevalence of malnutrition among CWDs was higher than that of children in general. Staying at institutions did not necessarily improve their malnutrition. The risks of different types of malnutrition differed by disability type and severity. Therefore, interventions should be tailored for specific types of disability (institutions or home). Particularly, the results suggest the importance of feeding support to prevent underweight and stunting among CWDs with physical and developmental disabilities. The results also suggested the importance of dietary diversity support to prevent underweight among CWDs with physical disabilities and nutrition control to prevent obesity and overweight among CWDs with developmental disabilities and autism.

This study had two strengths. First, it covered both under- and overnutrition in the CWD population, which has rarely been performed in LMICs. Second, malnutrition was assessed by disability type along with the place of accommodation in the Kathmandu Valley. However, this study also has limitations, such as missing data, no comparison group from the non-disabled children group, and community-dwelling CWDs. The sample size was limited, although this study enumerated all the institutions in the study site that permitted to conduct this study and recruited all eligible CWDs in these institutions. Moreover, malnutrition was assessed using only growth indicators; no biochemical tests or clinical procedures were performed. However, to address these limitations, further analysis was conducted to determine the association of independent variables with malnutrition among CWDs by including variables that had missing data in the regression model.

## Conclusion

In this study, almost two-thirds of CWDs displayed at least one form of malnutrition. Factors associated with malnutrition were identified: disability type, severity of a disability, feeding ability, age, parental status, and feeding diversity. However, accommodation type was not associated with malnutrition.

Monthly or quarterly screening for over- and undernutrition is recommended to reduce malnutrition. In addition, nutritional intervention programs should be developed and implemented in all institutions to improve nutritional status, considering the type and severity of a disability.

## Supplementary Information

**Additional file 1**

## Data Availability

All data collected and analyzed during this study are included in this published article [and its supplementary information files].

## Abbreviations

aOR: Adjusted Odds Ratio
BAZ: BMI for age z-score
BMI: Body Mass Index
CDDS: Child Dietary Diversity Index
CI: Confidence interval
CWDs: Children with disabilities
GLM: Generalized linear model
HAZ: Height for age z-score
KFN: Karuna Foundation Nepal
LMICs: Low- and middle-income countries
NDHS: Nepal Demographic and Health Survey
NFDN: National Federation of the Disabled-Nepal
RRR: Relative Risk Ratio
SD: Standard Deviation
US: United States
WAZ: Weight for age z-score
WHO: World Health Organization
