# Supplementary material for: Accommodation and disability-specific differences in nutritional status of children with disabilities in Kathmandu, Nepal: A cross-sectional study
Source: BMC Public Health. 2023 Feb 13;23:315. doi: 10.1186/s12889-023-14999-z (PMC9926754; doi:10.1186/s12889-023-14999-z)
Supplement: Supplementary file 1 — Additional file 1 [file 12889_2023_14999_MOESM1_ESM.zip › Supplimentary Files/Questionnaire for Malnutrition among children with disabilities-English.docx]

**Prevalence of malnutrition and factors associated with nutritional status of children with disabilities in Nepal: A comparative cross-sectional study**

Survey Questionnaire

Questionnaire No:

Hello. My name is _______________________________________. I am working with the research team from The University of Tokyo. We are conducting a research on Prevalence of malnutrition and factors associated with it among children with disabilities in Nepal. I would be appreciating your participation in this research. I would like to ask you about feeding habits of your child, disability and certain demographic characteristics related questions. I will also be measuring body weight and height of the child. This questionnaire will take the time of 15 minutes and for anthropometric measurement, another 10 minutes. I promise and assure you that the information obtained from you will be kept secret to maintain the confidentiality. No parts of this interview is being recorded.

Participation in this survey is voluntary and you can choose not to answer any individual question or all of the questions. However, I hope that you will participate in this survey since your views are important.

Do you have any questions?

May I begin the interview now?

Signature of the respondent …………………………………. Date: ………………………………..

**Respondent does not agree to give answers**

**Respondent agrees to give answers**

**End**

**Section 1: Background Characteristics**

| **Q No** | **Questions** | **Coding/Answers** | **Skip** |
| --- | --- | --- | --- |
| 1 | Number of respondents answering the question | One…………………………………….. 1  Two………………………………………2 |  |
| 2 | Relation of the respondent 1 with child | Self ……………………………………………… 1  Caretaker from institute ………………………… 2  Mother …………………………………………... 3  Caretaker from home ……………………………. 4 | If 3 or 4 go to QN 4 |
| 3 | Relation of the respondent 2 with child | Mother …………………………………….…….. 1  Caretaker from home ……………………………. 2 |  |
| 4 | Name of the Institute | ……………………………………………………….. |  |
| 5 | Address of the Institution | ………………..……Village ………… Ward No. …………………………….Rural/Municipality …………………………………District |  |
| 6 | What is the type of this institution?  (Type by functions) | Rehabilitation Centre ……………………….….. 1  Special School ……………………………..….… 2  School …………………………………………..... 3  Day care center …………………………….……. 4  Others ……………………………………………. 5 |  |
| 7 | What is the type of this institution?  (Type by Authority) | Government …………………………………….... 1  Community based ……………..…………………. 2  NGO/INGO ………………………………………. 3  Private …………………………………………….. 4  Others …………………………………………….. 5 |  |

**Section 2: Demographic Characteristics**

| 8 | Date of birth of the child  (DD-MM-YY) | …… day …… month ………… year (BS)  …… day …… month ………… year (AD) |  |
| --- | --- | --- | --- |
| 9 | Age of the child (in completed years) | ……………… years |  |
| 10 | Sex of the child | Male ……………………………………. 1  Female …………..…………………..…. 2  Others ….………………………….…… 3 |  |
| 11 | Where does the child sleep usually? | Home ………………………………..…. 1  Institution …………..………………..… 2  Others (Specify) ……..……………..….. 3 |  |
| 12 | Does child has disability? | Yes …………………………………….. 1  No ………….…………………………... 0 |  |
| 13 | What type of disability? | Physical disability …………………….. 1  Visual impairment (Blindness) …..…….. 2  Hearing impairment (Deafness) …………3  Hearing and visual disability (Deaf-blindness) ……………….………….…. 4  Speech or language impairment .…..…. 5  Mental and psychosocial disability ...… 6  Intellectual disability ……….………….. 7  Hereditary Hemophilia related disability ……………………………..……….……. 8  Autism …………………………….……. 9  Multiple disability ……....………….….. 10 |  |
| 14 | Does this child has disability ID Card? | Yes …………………..…………………..1  No ………………….……………………0 |  |
| 15 | What is the type of ID card? | Red …………………………………….. 1  Blue ……………………………………. 2  Yellow ………………………………… 3  White …………………………………. 4 |  |
| 16 | What is the mode of communication of this child? | Verbal …………………………………… 1  Non-verbal ……………..………………. 2 |  |

**Section 3: Maternal Characteristics**

| 17 | What is the current age of mother? (to calculate the age of mother while giving birth to this child) | ………………. Years |  |
| --- | --- | --- | --- |
| 18 | What is the schooling level of mother?^[[1]](#footnote-1)^ | No any school attended ………………… 0  Non-Formal education ………………….. 1  Primary school ………………………...... 2  Lower secondary school ……………….. 3  Secondary school ……………………….. 4  Higher secondary ……………………….. 5  University degree ……………..……....... 6 |  |
| 19 | This child was born at which place? | Hospital government ……………………. 1 Hospital private ……………………..….. 2  Primary Health Care Centre …………….. 3  Health Post …………………………… 4  Private clinic/Pharmacy ..……………. 5  Home ………………………………… 6  Road ………………………………… 7  Others …….…………………………. 8 |  |
| 20 | Does this child has exclusive breastfeeding for 6 months? ^[[2]](#footnote-2)^ | Yes ………………………………….. 1  No …………………………………… 0 |  |
| 21 | How many months does this child breastfed? | …………….. months |  |

**Section 4: Feeding Characteristics**

| 22 | Does this child has any difficulty while feeding at perinatal period?  (after birth to till 7 days) | Yes ……………………………………… 1  No ………………………………………. 0  Don’t remember ……………………...... 9 |  |
| --- | --- | --- | --- |
| 23 | What is the feeding ability of this child? | Feeds self ……………………………… 1  Has to be fed ….……………………… 2 |  |
| 24 | What is the time given by caretaker to feed in one time? | …………….. minutes |  |
| 25 | How many times does this child eat in a day (24 hours)? (Regardless of the place) | ……………… times |  |
| 26 | How many times does this child eat in this institution? | ……………… times |  |
| 27 | Where does this child eat breakfast today? | Home …………….……………………. 1  Institution …………………………….. 2  Others (specify) .....…………………..… 3  Not ate …………………………………. 0 |  |

**Section 5: Child Consumption**

| **Food Groups** | **24 Hour Recall** | | **7 Days Recall** | |
| --- | --- | --- | --- | --- |
|  | **Consumed over the last 24 hours (1=Yes; 0=No)** | **If Yes, number of times** | **Consumed over the last 7 days** | **If Yes, number of times** |
| **Cereals and cereal products** (Rice, Chapatti) |  |  |  |  |
| **Tubers and roots**  (Potatoes, Yam, or foods made from roots. |  |  |  |  |
| **Vitamin A rich vegetables and fruits**  e.g. Pumpkins, carrots, orange sweet potatoes, ripe mangoes, papayas + other locally available vitamin A rich fruits and vegetables |  |  |  |  |
| **Other vegetables including wild ones** |  |  |  |  |
| **Other fruits including wild ones** |  |  |  |  |
| **Pulses/legumes/nuts** e.g. beans, peas, lentils, green grams etc |  |  |  |  |
| **Meat and meat products** |  |  |  |  |
| **Eggs** |  |  |  |  |
| **Fish** |  |  |  |  |
| **Milk and dairy products** |  |  |  |  |
| **Oils/Fat** |  |  |  |  |
| **Baked goods** (Cake, Cookies, Biscuit, Noodle) |  |  |  |  |
| **Tea with sugar/ honey** |  |  |  |  |

**Section 6: Anthropometry**

| **No.** | **Questions** | **Coding/Answer** | **Remarks** |
| --- | --- | --- | --- |
| 1 | Height in centimeters  (to the nearest 0.1cm) |  |  |
| 2 | Weight in KG  (to the nearest 0.1kg) |  |  |
| 3 | MUAC  (to the nearest 0.1cm) |  |  |

1. Primary school: Class 1-5; Lower secondary school: Class 6-8; Secondary school: Class 9-10; Higher secondary school: Class 11-12; University degree: Bachelor’s degree and more. [↑](#footnote-ref-1)
2. Exclusive Breastfeeding is defined as feeding mother’s breast milk only to baby for six months without even water, liquid or food. [↑](#footnote-ref-2)
