# Supplementary material for: Accommodation and disability-specific differences in nutritional status of children with disabilities in Kathmandu, Nepal: A cross-sectional study
Source: BMC Public Health. 2023 Feb 13;23:315. doi: 10.1186/s12889-023-14999-z (PMC9926754; doi:10.1186/s12889-023-14999-z)
Supplement: Supplementary file 1 — Additional file 1 [file 12889_2023_14999_MOESM1_ESM.zip › Supplimentary Files/Questionnaire for Malnutrition among children with disabilities-Nepali.docx]

**ckfËtf ePsf afnafnLsfx?sf] s'kf]if0fsf] cj:yf / plgx?sf] kf]if0fnfO{ k|efj kfg]{ tTjx?sf] cWoog**

;e{]If0f k|ZgfjnL

k|Zg g=:

**v08 s M cfwf/e"t ljj/0f**

| **k\|Zg g+=** | **k\|Zgx?** | **pQ/x? tyf sf]l8ª** | **l:sk** |
| --- | --- | --- | --- |
| ! | pQ/bftfx?sf] hDdf ;+Vof | Ps………………………………………..…….. !  b'O{……………………………..…………………@ |  |
| @ | pQ/bftf ! sf] aRrf;+usf] ;DaGw s] xf] < | :j+od …………………………………………… !  ;+:yfsf] /]vb]v ug]{ JoQmL …………………… @  cfdf ………………..……………….…………... #  3/sf] /]vb]v ug]{ JoQmL ………………..….…. $ | olb pQ/ # / $ eP k\|Zg g+=$ df hfg] |
| # | pQ/bftf @ sf] aRrf;+usf] ;DaGw s] xf] < | cfdf …………………………………….…….. !  3/sf] /]vb]v ug]{ JoQmL ……………………. @ |  |
| $ | aRrf a:g] jf k9\g] ;+:yfsf] gfd s] xf] < | ……………………………………………………….. |  |
| % | ;+:yfsf] 7]ufgf | ………………..……6f]n ………… j8f g+= …………………………….gu/kfnLsf÷ufpFkfnLsf …………………………………lhNnf |  |
| ^ | o; ;+:yf s'g k\|sf/sf] xf] <  -;+:yfsf] sfo{sf lx;fan]_ | k'g:yf{kgf s]Gb\| ………………………….….. !  laz]if :s'n ………………………….....….… @  laBfno ……………………………………..... #  lbjf x]/rfxf s]Gb\| …………………..….……. $  cGo ………………………………..…………. % |  |
| & | o; ;+:yf s'g k\|sf/sf] xf] <  -;f+u7gLs jf nufgLsf lx;fan]_ | ;/sf/L …………………………………….... !  ;fd'bfO{s…………….…..………………….. @  u}/ ;/sf/L ;+:yf ………………….………. #  lghL …………………………………….….. $  cGo ……………………………………...….. % |  |

**v08 v M hg;fª\vLsL ljj/0f**

| * | aRrfsf] hGdldtL slxn] xf] <  -u–d–;f_ | =====ut] ======== dlxgf ======= ;fn -lj=;_  =====ut] ======== dlxgf ======= ;fn -O{=;_ |  |
| --- | --- | --- | --- |
| ( | aRrfsf] pd]/ slt xf] < -k'/f ePsf] aif{df_ | ======================aif{ |  |
| !) | aRrfsf] lnË s'g xf] < | k'?if ====================================== !  dlxnf ==================================== @  cGo ======================================= # |  |
| !! | w]/}h;f] ;do aRrf sxfF ;'T5 < | 3/df ======================================== !  ;+:yfdf ==================================== @  cGo ========================================= # |  |
| !@ | s] aRrfdf ckfËtf 5 < | 5 ============================================= !  5}g =========================================== ) | olb 5}g eg] k\|Zg g+ !^ df hfg] . |
| !# | aRrfnfO{ s'g k\|sf/sf] ckfËtf 5 < | Zff/Ll/s==================================== !  b[li6========================================= @  ;'gfO{======================================== #  >j0fb[i6ljlxg============================ $  :j/ tyf jf]nfO{========================== %  Dfgl;s jf dgf];fdflhs ckfËtf=== ^  Aff}l¢s======================================= &  x]d]lkmlnof================================== *  cl6Hfd====================================== (  ax' ckfËtf================================= !) |  |
| !$ | s] o; aRrfsf] ckfËtf kl/ro kq 5 < | 5 ===============================!  5}g ============================ ) |  |
| !% | ckfËtf kl/ro kq s'g k\|sf/sf] xf] < | /ftf] ==================================== !  lgnf] =================================== @  kFx]nf] =================================== #  ;]tf] ==================================== $ |  |
| !^ | o; aRrfsf] ;+rf/ ug]{ t/Lsf jf dfWod s] xf] < | df}lvs jf af]n]/ ===================== !  ;f+s]tLs efiff ======================== @ |  |

**v08 u M dft[:jf:Yo ljj/0f**

| !& | cfdfsf] xfnsf] pd]/ stL xf] < -k'/f ePsf] aif{df n]Vg'xf];_ | ====================== aif{ |  |
| --- | --- | --- | --- |
| !* | cfdfsf] ;}lIfs of]Uotf stL xf] <^[[1]](#footnote-1)^ | n]vk9 ug{ g;Sg] ===================== )  k\|f}9 lzIff ================================ !  k\|fylds lzIff ========================== @  lgDg dfWoflds lzIff ================ #  dfWoflds lzIff ======================== $  pRr dfWoflds lzIff ================= %  ljZjlaBfno lzIff ==================== ^ |  |
| !( | aRRfsf] hGd s'g 7fFpdf ePsf] lyof]< | ;/sf/L c:ktfn ====================== !  lghL c:ktfn ========================= @  k\|fylds :jf:Yo s]G› ================ #  x]Ny kf]:6 ============================== $  SnLlgs÷kmfd]{;L ====================== %  3/ ======================================== ^  af6f] ===================================== &  cGo ====================================== * |  |
| @) | s] o; afns÷afnLsfn] ^ dlxgf;Dd cfdfsf] b'w dfq} vfPsf] xf] -k'0f{ :tgkfg_ ? ^[[2]](#footnote-2)^ | xf] ========================================!  xf]O{g =================================== @ |  |
| @! | stL ;do;Dd o; afns÷afnLsfn] cfdfsf] b'w vfPsf] xf]< | ========================== dlxgf |  |

**v08 u M vfgkLg ;DaGwL hfgsf/L**

| @@ | s] o; afns÷aflnsfnfO{ hGd]sf] & lbg ;Dd cfdfsf] b'w r':g jf b'w vfg ;d:of k/]sf] lyof] < | lyof] =========================== !  lyPg ========================= @  yfxf 5}g ===================== ( |  |
| --- | --- | --- | --- |
| @# | s] o; afns÷aflnsfn] vfgf cfFkm} vfG5 < | cfFkm} vfG5 =========================== !  c?n] v'jfpg' k5{ =================== @ |  |
| @$ | cleefjsn] Ps k6sdf vfgf v'jfFpbf slt ;do lbg'x'G5 < | ===================== ldg]6 |  |
| @% | o; afns÷afnLsfn] lbg e/Ldf -@$ 306f_ stL k6s vfgf vfG5g < -;a} 7fFpdf_ | ===================== k6s |  |
| @^ | o; afns÷afnLsfn] lbg e/Ldf -@$ 306f_ o; ;+:yfdf stL k6s vfgf vfG5g < | ===================== k6s |  |
| @& | o; afns÷afnLsfn] cfh laxfgsf] vfhf sfFxf vfP < | 3/ ==================================== !  ;+:yf ================================= @  cGo -v'nfpg'xf];_ ================ #  vfPsf] 5}g ========================== ) |  |

**v08 % M afns÷afnLsfsf] vfB pkef]u**

| **vfBfGosf] ;d'x** | **@$ 306fsf] ;Demgf** | | **& lbgsf] ;Demgf** | |
| --- | --- | --- | --- | --- |
|  | **lat]sf] @$ 306fdf vfPsf] -!Ö5, )Ö5}g_** | **olb xf] eg], slt k6s** | **lat]sf] & lbgdf vfPsf] -!Ö5, )Ö5}g_** | **olb xf] eg], slt k6s** |
| **cGg tyf cGgaf6 ag]sf kl/sf/x?** -eft, /f]6L_ |  |  |  |  |
| **h/f tyf 6\o'a/** -cfn', t?n, jf cGo h/fhGo vfB j:t'_ |  |  |  |  |
| **le6fldg P kfO{g] t/sf/L tyf kmnkm'n**  -km;L{, ufh/, ;'Gtnf, ;v/v08, cfFk, d]jf cflb_ |  |  |  |  |
| **h+unL t/sf/L tyf cGo t/sf/Lx?** |  |  |  |  |
| **h+unL kmnkm'n tyf cGo kmnkm'nx?** |  |  |  |  |
| **bfn tyf u]8fu'8L** |  |  |  |  |
| **Df;' tyf df;'hGo kbf{y** |  |  |  |  |
| **c08f** |  |  |  |  |
| **df5f** |  |  |  |  |
| **b'w tyf b'whGo kbf{y** |  |  |  |  |
| **t]n tyf £o"** |  |  |  |  |
| **a]s/L** -s]s, s'lsh, la:s'6, rfprfp cflb _ |  |  |  |  |
| **lrlg jf dx xfn]sf] lrof** |  |  |  |  |

**v08 ^ M prfO{ tyf tf}n dfkg**

| **g+=** | **k\|Zgx?** | **pQ/x?÷sf]8Lª** | **s}lkmot** |
| --- | --- | --- | --- |
| ! | prfO{ -;]=dL=_  -)=! ;]=dL=sf] glhs_ |  |  |
| @ | tf}n -s]=hL=_  -)=! s]=hL=sf] glhs_ |  |  |
| # | MUAC  -)=! ;]=dL=sf] glhs_ |  |  |

1. k|fylds laBfnoM sIff !–%, lgDg dfWodLs laBfnoM ^–*, dfWodLs laBfnoM (–!), pRr dfWodLs laBfnoM !!–!@,ljZjlaBfnoM :gftsf]Q/ jf ;f] eGbf dfyL [↑](#footnote-ref-1)
2. k'0f{ :tgkfg eGgfn] aRrfn] ^ dlxgf;Dd cfdfsf] b'w dq} vfPsf] eGg] a'lemG5 . o;df aRrfn] b'w afx]s cGo vfg]s'/f, emf]n kbfy{ tyf kfgL klg gvfPsf] a'lemG5 . [↑](#footnote-ref-2)
